# The Role of Procalcitonin in the Diagnosis of Meningitis: A Literature Review

**DOI:** 10.3390/jcm7060148

**Published:** 2018-06-11

**Authors:** Dimitrios Velissaris, Martina Pintea, Nikolaos Pantzaris, Eirini Spatha, Vassilios Karamouzos, Charalampos Pierrakos, Menelaos Karanikolas

**Affiliations:** 1 Department of Medicine, University of Patras, 26504 Patras, Greece; dimitrisvelissaris@yahoo.com; 2 University of Medicine and Pharmacy, 400337 Cluj-Napoca, România; martina.pintea@yahoo.com; 3 School of Medicine University of Patras, 26500 Patras, Greece; npantzaris@gmail.com (N.P.); eirini.spatha@outlook.com (E.S.); 4 Intensive Care Unit, University Hospital of Patras, Rion 26500, Greece; karamouzos@hotmail.com; 5 Intensive Care Department, Brugmann University Hospital, 1020 Brussels, Belgium; charalampos.pierrakos@chu-brugmann.be; 6 Department of Anesthesiology, Washington University School of Medicine, St. Louis, MO 63144, USA

**Keywords:** procalcitonin, bacterial meningitis, viral meningitis, antibiotic therapy, biomarker, differential diagnosis

## Abstract

Objective: To review the current published literature on the use of procalcitonin as a diagnostic and prognostic marker in adult patients with meningitis. Methods: We conducted a PubMed search to identify all relevant publications regarding the diagnostic and prognostic value of serum procalcitonin in patients with a known or suspected central nervous system infection. We also reviewed the bibliographies of all identified manuscripts in an attempt to identify additional relevant references. Results: A significant body of evidence suggests that serum procalcitonin has a promising role and can be a useful biomarker in the assessment of patients with meningitis. Conclusions: Our literature review suggests that data on the role of Cerebrospinal Fluid (CSF) procalcitonin are limited, whereas serum procalcitonin (S–PCT) is probably a useful tool in the evaluation of patients with a known or suspected central nervous system infection and can help distinguish between bacterial and viral meningitis.

## 1. Introduction

Meningitis is a serious medical condition and can be a major cause of morbidity and mortality. Early diagnosis and timely initiation of appropriate antibiotic therapy is crucial for reducing mortality from bacterial meningitis.

The term “biomarker”, as used in daily clinical practice, refers to molecules and biological products used as markers for the assessment of disease progression or as indicators for the presence of an abnormal clinical state. Biomarkers can be specific cells or genes, gene products, enzymes or hormones, have characteristic defined biological properties, and can be detected and measured in biological fluids (plasma, serum, cerebrospinal fluid, bronchoalveolar lavage) or body tissues. More than 178 biomarkers have been identified in the field of sepsis, but none seem to have sufficient specificity or sensitivity for routine use in daily clinical practice [1] and some require considerable time, effort, and costs to measure. In addition, the reliability and validity of certain proposed biomarkers have not been thoroughly tested [2]. Procalcitonin (PCT) and C-reactive Protein (CRP) are the biomarkers most commonly used, but have a limited ability to distinguish sepsis from other inflammatory and non-inflammatory states or to predict outcomes.

Serum procalcitonin (S–PCT) has been used as biomarker in sepsis because S–PCT levels are elevated in bacterial, parasitic, or fungal infections, while they remain normal or only slightly elevated in viral infections. Because early recognition of viral versus bacterial meningitis is critical for the prompt initiation of treatment to improve prognosis, a reliable method distinguishing bacterial from viral meningitis could help clinicians limit inappropriate antibiotic treatment. This review was conducted to evaluate the current knowledge on the use of S–PCT as a tool for the diagnosis of meningitis and for distinguishing bacterial meningitis (BM) from viral meningitis (VM).

## 2. Methods

To identify relevant publications of interest, we conducted a PubMed search on 24 May 2018 using the terms ‘procalcitonin and meningitis’ as “Title/Abstract” or as “MeSH Terms”. Because “Procalcitonin” is not a “Mesh Term” in PubMed, we also used the term “Calcitonin” in “Mesh Terms” during the search. The structure of the search in the “Search details” window of the PubMed website was (procalcitonin [Title/Abstract] OR “calcitonin” [MeSH Terms]) AND (meningitis [Title/Abstract] OR “meningitis” [MeSH Terms]). For the purposes of this review, we then limited the search to “Humans” and only considered manuscripts presenting data on adults. We also reviewed the bibliographies of all identified manuscripts to identify additional relevant publications. For the purposes of this review, we included all types of publications, including case reports, case series, and review articles, regardless of publication date. Publications in languages other than English were included only if they had a meaningful detailed abstract in English.

## 3. Results

The PubMed literature search generated 157 references, but the number of references was reduced to 125 after limiting the search to “Humans”. After further evaluation, 38 publications were included in this review.

In a prospective study published in 1999, Viallon et al. evaluated 105 emergency department patients admitted with suspected meningitis. Based on clinical findings, gram staining, cultures, and Cerebrospinal Fluid (CSF) chemical analysis, 23 patients had bacterial meningitis, 57 had viral meningitis, and meningitis was ruled out in 25 patients. Although two patients with previous antibiotic therapy had S–PCT levels of <0.5, S–PCT was the best marker for differentiating between bacterial and viral meningitis: Using S–PCT of >0.2 ng/mL as the threshold, S–PCT sensitivity and specificity approaches 100% for the diagnosis of acute bacterial meningitis [3].

A prospective study published by Schwarz in 2000 included 30 patients with meningitis (16 with acute bacterial and 14 with abacterial meningitis) and assessed whether S–PCT levels were elevated in patients with bacterial meningitis. Results of the study showed that, although false negative results can occur, S–PCT is a useful variable for distinguishing bacterial from non-bacterial meningitis: Because S–PCT levels do not increase in cases of viral meningitis, even with viral sepsis, increased S–PCT levels indicated bacterial origins of an infection with high specificity [4].

In 2000, Viallon et al. published the results of a prospective study on 179 patients admitted to the emergency department on suspicion of meningitis. Of those, 32 patients had bacterial meningitis and 90 had viral meningitis, whereas 57 patients did not have meningitis. The authors assessed the role of CSF parameters (cytology, protein, glucose, lactate) and serum parameters (CRP, S–PCT) for differentiating between bacterial and viral meningitis, and demonstrated that S–PCT was the most discriminant variable, using a threshold value of 0.93 ng/mL in their population [5].

Shimetani et al. reported extremely high CSF CRP levels in patients with bacterial meningitis, but only in 10% of patients with viral meningitis. Among patients with bacterial meningitis, S–PCT levels were more elevated in those with a more serious infection. PCT levels in CSF did not differ significantly between patients with bacterial, viral, or mycotic meningitis. However, S–PCT levels were very high in all bacterial meningitis patients, especially in the most serious cases [6].

A report by Hoffmann et al., published in 2001, assessed S–PCT levels in 12 adult patients with meningitis and suggested that S–PCT has limited diagnostic value in adults with bacterial meningitis, especially in cases with unusual agents or nosocomial origin. Increased S–PCT levels in bacterial meningitis may indicate the presence of bacterial inflammation outside the Central Nervous System (CNS) [7].

A review by O’Connor published in 2001 included manuscripts published from 1990–2001 to assess the diagnostic usefulness of S–PCT in critical illness. This publication concluded that, although there is debate regarding the superiority of S–PCT as a sepsis biomarker compared to other biomarkers, a number of studies support the usefulness of S–PCT in differentiating between bacterial and viral meningitis [8].

A prospective study on 45 adult patients with CNS infection (20 with bacterial meningitis and 25 with tick-borne encephalitis), published in 2001, evaluated the role of S–PCT and CSF procalcitonin in differentiating acute bacterial vs. viral meningitis. Median S–PCT level was 6.45 ng/mL (0.25–43.76 ng/mL) in patients with bacterial meningitis vs. 0.27 ng/mL (0.05–0.44 ng/mL) in patients with viral meningitis, and the authors concluded that S–PCT and CSF PCT concentrations >0.5 ng/mL seem to be reliable indicator of bacterial CNS infection [9].

A study by Martinez et al. in 2002 attempted to evaluate the role of S–PCT monitoring in the differential diagnosis of ventriculitis in adult Intensive Care Unit (ICU) patients. The study included 15 consecutive ICU patients with ventriculitis and a ventricular catheter in place, and compared these data with 10 patients with community-acquired bacterial meningitis. The authors concluded that, in contrast to bacterial meningitis, monitoring of S–PCT alone is not helpful for the diagnosis of ventriculitis [10].

A case report published in 2004 presented the case of a 73-year-old woman with progressively worsening headaches, nausea, vomiting, and neck stiffness. As her clinical condition deteriorated, she developed diffuse brain edema and hydrocephalus, requiring external ventricular drainage (EVD) and admission to a neurologic ICU, with a subsequent diagnosis of severe post-myelographic chemical meningitis. The authors compared CSF and serum inflammatory markers of this patient versus seven patients with proven bacterial meningitis and concluded that S–PCT may be useful in differentiating between bacterial and chemical causes of CNS inflammation [11].

A study by Kepa et al. assessed the role of CSF PCT and S–PCT levels in the differential diagnosis of adults with CNS infections. The study included 17 patients with bacterial meningoencephalitis and 16 patients with lymphocytic meningitis and showed that CSF and plasma PCT levels were significantly different between these two patient groups. These results supported the usefulness of measuring plasma PCT levels in the differential diagnosis of CNS infections in adults. With regards to the role of CSF PCT, the authors concluded that CSF PCT levels are less important for differential diagnosis, but correlate with the severity of bacterial meningoencephalitis and can be taken into consideration when predicting prognosis and outcomes [12].

In 2005, a study by Viallon et al. described the change in S–PCT levels during the treatment of 48 patients with community-acquired acute bacterial meningitis. Bacterial infection was documented in 45 patients and initial antibiotic treatment was effective in all patients. Serum PCT levels were measured on admission and on day two, and showed that S–PCT levels decline rapidly with appropriate antibiotic therapy. The authors concluded that the rapid decline of S–PCT levels reduces the value of performing lumbar puncture 48 to 72 h after admission to assess the effectiveness of antibiotic therapy [13].

Ernst and colleagues evaluated serum and CSF procalcitonin levels in patients with dementia disorders and neuro-inflammation. The study included 40 patients with probable Alzheimer’s disease, 12 patients with frontotemporal dementia (FTD), 8 patients with dementia with Lewy bodies (DLB), 12 patients with vascular dementia (VD), 16 patients with acute neuroinflammation, and 50 non-dementia control patients (18 surgery patients and 32 patients with other neurologic diseases)and showed that, compared to non-dementia controls, CSF procalcitonin levels were increased in patients with dementia diseases and acute neuro-inflammation. In addition, in matched serum samples, S–PCT levels were elevated in meningitis patients, but not in dementia patients [14].

An observational cohort study by Knudsen et al. included 55 patients with suspected meningitis and compared the diagnostic value of serum sCD163 levels, CRP, and procalcitonin in bacterial infection and meningitis and showed that, although elevated serum sCD163 levels seem to be the most specific biomarker for differentiating between bacterial and non-bacterial disease (specificity 0.91; sensitivity 0.47), the overall diagnostic accuracy of CRP (Area Under the Curve (AUC) = 0.91) and PCT (AUC = 0.87) were superior compared to sCD163. The authors concluded that, although PCT and CRP had very high accuracy for distinguishing between bacterial and viral infection, none of them were useful as an independent tool for diagnosis in patients presenting with purulent meningitis [15].

A prospective multicenter study, published in 2007, included 151 patients with bacterial or nonbacterial meningitis and negative initial Gram stains from three teaching hospitals in France and reported laboratory data, including results of CSF analysis (CSF leukocyte count, percentage of CSF leukocyte, CSF/blood glucose ratio, CSF protein), serum CRP, and serum PCT, together with clinical findings and outcomes. The study evaluated the accuracy of laboratory results in differentiating between bacterial and non-bacterial meningitis in patients with meningitis and a negative gram stain, and concluded that CSF laboratory results have some role in distinguishing bacterial from non-bacterial meningitis, whereas serum CRP (AUC 0.81 (95% CI 0.58–0.92) and S–PCT levels (AUC 0.98, 95% CI, 0.83–1.00) are excellent predictors of bacterial meningitis, with S CPT being clearly superior (*p* < 0.05) [16].

A prospective study from the Saint-Etienne University Hospital in France collected data from all patients admitted to the emergency unit with suspected meningitis between 1997 and 2009. Data were collected on 97 patients with bacterial meningitis and 218 patients with viral meningitis, but, after 62 patients with Bacterial Meningitis (BM) were excluded for various reasons, the study only included data from 35 patients with BM and negative direct CSF examination. The aim of the study was to determine the ability of several parameters used for the diagnosis of acute meningitis in differentiating between bacterial and viral meningitis in adult patients with a negative CSF examination. In this study, S–PCT had a 95% sensitivity, 100% specificity, and 100% negative predictive value, as well as a 97% positive predictive value for distinguishing BM versus Viral Meningitis (VM) when using a diagnostic cut-off level of 0.28 ng/mL (AUC, 0.99; 95% CI, 0.99 to 1) [17].

A prospective study on 36 adult patients with acute meningitis was published in 2012. The aim of the study was to evaluate the role of serum procalcitonin levels over time during treatment for central nervous system infections. Serum procalcitonin levels were measured before the initiation of treatment and 24 and 72 h after treatment started. Results showed that mean PCT levels were higher in patients who did not improve and that the reduction of serum PCT levels were more significant after 72 h in patients who improved. The authors emphasized the role of serum PCT levels as a marker for follow up in treating patients with bacterial meningitis [18].

A study by Choi assessed the value of serum procalcitonin in differentiating post-operative bacterial meningitis (PBM) versus postoperative aseptic meningitis (PAM) after neurological surgery and included patients who had cerebrospinal fluid pleocytosis within 14 days of surgery. The study compared PCT in 14 patients with PBM against 64 patients with PAM and showed that serum PCT had limited value for diagnosing PBM and serum PCT levels of ≥0.15 ng/mL had 80% specificity. However, CPT combined with other biomarkers can be a useful adjunct for increasing diagnostic sensitivity [19].

An observational study by Tian et al., from Guadong, China and published in 2014, investigated the value of procalcitonin in the discrimination between sepsis and systemic inflammatory response syndrome (SIRS). The study included patients treated in a neurological intensive care unit and serum levels of C-reactive protein and S–PCT were evaluated on admission day, on the day of diagnosis of SIRS or sepsis, and on days three and seven after the diagnosis. Results of the study showed significant differences in S–PCT levels between groups at all stages of sepsis. The authors concluded that S–PCT has significant value as an index for discriminating sepsis from SIRS and in determining sepsis severity [20].

A study by Abdelkader et al. published in 2014 evaluated 40 patients with suspected acute meningitis and negative gram stains compared to 10 healthy controls. The goal of the study was to evaluate the role of S–PCT in differentiating bacterial from aseptic meningitis in patients with negative cerebrospinal fluid (CSF) examination on admission and after three days of treatment, and to assess the role of PCT and other inflammatory markers in relation to treatment efficacy. In this study, patients in the bacterial group had significantly higher serum PCT on admission compared to the aseptic group (2.49 ± 2.54 vs. 0.89 ± 0.69, *p* < 0. 001), and there was a significant difference in bacterial versus. aseptic meningitis, even after three days of treatment (1.70 ± 1.58 vs. 0.64 ± 0.51, *p* < 0.001) [21].

A prospective observational study, published by Shen et al. in 2015, assessed the diagnostic value of serum and CSF PCT levels in 120 patients with meningitis-like symptoms and showed that both S–PCT and CSF PCT levels were increased in patients with bacterial meningitis (BM). The area under the Receiver Operator Characteristic (ROC) curve was 0.96 (CI 0.93–1.00) for S–PCT versus 0.9 (CI 0.83–0.96) for CSF PCT in the diagnosis of BM. When using 0.88 ng/mL as a threshold, S–PCT had an 87% sensitivity and 100% specificity for the diagnosis of BM. The study concluded that both S–PCT and CSF PCT have value for the diagnosis of BM, but the diagnostic value of S–PCT is superior [22].

In another prospective observational study, Omar et al. collected data on CRP, S–PCT, and CSF cultures every other day in 36 adult patients with severe head trauma and ventriculostomy, and observed elevated S–PCT concentration in all five patients who developed ventriculostomy-related infections. Mean serum PCT was <2.0 ng/mL in patients with negative CSF cultures versus 4.18 ng/mL in patients with positive cultures. The study concluded that an early increase of S–PCT levels is a valid indicator of bacterial CNS infection in patients with head trauma and External Ventricular Drainage (EVD) [23].

A retrospective clinical study by Li et al. assessed the diagnostic value of CSF procalcitonin combined with CSF lactate levels in distinguishing post-neurosurgical bacterial meningitis (PNBM) from aseptic meningitis in 178 hospitalized patients with suspected PNMB (50 patients with PNBM vs. 128 patients without PNBM). Median (min, max) CSF procalcitonin levels were 0.2 (0–3.1) in patients with PNBM versus 0 (0–0.5) in patients with non-PNBM (*p* < 0.001), and ROC analysis revealed a cut-off value of 0.075 ng/mL (AUC = 0.746, sensitivity 68.0%; specificity 72.7%, *p* < 0.001) for CSF procalcitonin. Similarly, median (min, max) CSF lactate levels were 5.3 (2.2–10.6) in patients with PNBM versus 2.3 (1.2–5.4) in patients with non-PNBM (*p* < 0.001), and ROC analysis revealed a cut-off value of 3.45 mmol/L (AUC = 0.943, sensitivity 90.0%; specificity 84.4%, *p* < 0.001) for CSF lactate. The study showed that PNBM patients have significantly higher CSF procalcitonin and CSF lactate levels compared with non-PNBM patients and concluded that CSF lactate and PCT levels have significant diagnostic value for PNBM, and could be useful in differentiating PNBM from non-PNBM [24].

A publication by Konstantinidis et al. in 2015 evaluated CSF procalcitonin levels and compared CSF procalcitonin levels with CSF levels of other established markers of infection, such as CRP, high-sensitivity CRP, and White Blood Cells (WBC) in 30 ICU, Medicine, Neurology, Hematology, and Pediatric patients with bacterial (*n* = 19) or viral (*n* = 11) meningitis, and in 28 patients with non-infectious diseases. In this study, CSF PCT levels were 4.714 ± 1.59 ng/mL in bacterial meningitis versus 0.1327 ± 0.03 ng/mL in patients with viral meningitis versus <0.1 ng/mL in patients with non-infectious diseases, with the authors concluding that S–PCT can be helpful in distinguishing bacterial meningitis from viral meningitis and other noninfectious CNS diseases [25].

A meta-analysis published in 2015 by Vikse et al. included nine studies with a total of 725 patients and concluded that serum procalcitonin had a pooled sensitivity of 0.90 (95% CI 0.84–0.94), a specificity of 0.98 (0.97–0.99), a positive likelihood ratio of 27.3 (8.2–91.1), a negative likelihood ratio of 0.13 (0.07–0.26), a diagnostic odds ratio of 287.0 (58.5–1409.0), and, thus, is far superior to CRP for rapid differentiation between bacterial and viral meningitis [26].

Kim et al. compared S–PCT levels in 26 patients with tuberculosis meningitis versus 70 patients with BM and 49 patients with VM in a retrospective study and showed that low S–PCT levels (≤1.27 ng/mL) independently distinguished tuberculosis meningitis from bacterial meningitis, with a 96.2% sensitivity and a 62.9% specificity. However, S–PCT levels were not significantly different in patients with tuberculosis versus viral meningitis. Logistic regression showed that an S–PCT level of >0.4 ng/mL was an independent predictor of a poor prognosis in patients with tuberculosis meningitis and had a negative correlation with Glasgow Coma Scale (GCS) scores at discharge (*r* = 0.437, *p* = 0.026) [27].

A prospective observational study published by Morales-Casado et al. in 2016 assessed the role of 32 clinical and epidemiological variables as predictors of bacterial meningitis in 154 patients aged over 15 years who presented in the Emergency Department with symptoms of acute meningitis. Multivariate logistic regression showed that four variables (S–PCT, CSF lactate ≥33 mg/dL, CSF glucose <60% of blood value, and CSF polymorphonuclears ≥50%) were excellent tools for the prediction of bacterial meningitis; the model using S–PCT ≥0.8 ng/mL and CSF lactate ≥33 mg/dL had an AUC of 0.992, with a 99% sensitivity and a 98% specificity for predicting bacterial meningitis (95% CI: 0.979–1; *p* < 001) [28].

Another prospective observational study by Morales-Casado et al. evaluated the usefulness of inflammatory markers for the diagnosis of bacterial meningitis in 220 patients and showed that S–PCT had the highest AUC (0.972; 95% CI, 0.946–0.998; *p* < 001) for the diagnosis of BM. Using 0.52 ng/mL as a cutoff, S–PCT had 93% sensitivity and 86% specificity for the diagnosis of BM overall, but sensitivity was 96% and specificity was 75% in patients >75 years old [29].

In 2016, Wei and colleagues published a systematic review and meta-analysis on the role of procalcitonin in the diagnosis of bacterial versus non-bacterial meningitis. The review included twenty-two studies with a total of 2058 patients; diagnostic accuracy of S–PCT and CSF PCT was assessed using the bivariate model and analysis showed that PCT is a useful biomarker for the diagnosis of bacterial meningitis: CSF PCT had 0.86 specificity and 0.8 sensitivity, whereas S–PCT had 0.97 specificity and 0.95 sensitivity [30].

A prospective, observational study published in 2016 by Morales Casado et al. evaluated serum PCT and C-reactive protein as markers for detection of bacterial meningitis in 98 patients diagnosed with acute meningitis in the emergency department (38 pts with BM, 33 with VM, 15 with probable VM, and 12 with presumptively diagnosed, partially treated acute meningitis). Data analysis showed that S–PCT levels were significantly higher in patients with BM (11.47 ± 7.76 ng/mL vs. 0.10 ± 0.15 ng/mL in viral meningitis, *p* < 0.001). Using 1.1 ng/mL as cutoff, S–PCT as diagnostic tool achieved 94.6% sensitivity, 72.4% specificity, 95.4% NPV, and 69.2% PPV, and AUC was 0.965 (95% CI, 0.921–1; *p* < 0.001). Based on these results, the authors concluded that S–PCT performs better than CRP in the detection of bacterial meningitis [31].

In a prospective observational study published in 2017, Zhang et al. measured S–PCT and CSF PCT, high-sensitivity C-reactive proteins (Hs-CRP), proteins, chloride, and glucose in three patient groups: 24 patients with suppurative meningitis, 20 with VM, and 22 with tuberculous meningitis (TBM). S–PCT values were significantly higher in the suppurative meningitis group, but declined significantly in suppurative meningitis patients after 72 h and seven days of treatment. In addition, admission CSF PCT levels were significantly lower in VM compared to TBM and suppurative meningitis patients, but CSF PCT values did not change significantly with treatment. The authors concluded that S–PCT changes over time can be useful in evaluating disease progression and response to treatment in patients with suppurative meningitis [32].

A retrospective clinical study on 80 patients with BM and 58 with VM, published by Park in 2017, showed that S–PCT >0.12 ng/mL is a significant marker for differentiating BM from VM, and also that S–PCT levels >7.26 ng/mL are associated with higher risk of death (OR = 9.09, 95% CI: 1.74–47.12, *p* = 0.016) [33].

Last, a prospective observational study published by Li et al. in 2017 included 143 ICU patients (49 with BM, 25 with TBM, 34 with viral meningitis/encephalitis (VM/E), 15 with autoimmune encephalitis (AIE), and 20 with non-inflammatory nervous system diseases (NINSD) to assess the value of CSF PCT, S–PCT and other biomarkers in the diagnosis of BM. In this study, CSF PCT levels (median, range) were significantly (*P* < 0.01) higher in BM patients (0.22, 0.13–0.54 ng/mL) compared to TBM (0.12, 0.07–0.16 ng/mL), VM/E (0.09, 0.07–0.11 ng/mL), AIE (0.06, 0.05–0.10 ng/mL), or NINSD (0.07, 0.06–0.08 ng/mL) patients. Furthermore, CSF PCT had the highest area under the receiver operating characteristic curve (AUROC) (0.881; 95% CI 0.810–0.932; cutoff 0.15 ng/mL; sensitivity 69.39%; specificity 91.49%), whereas S–PCT was less useful (AUROC 0.759, 95% CI 0.669–0.849, cutoff 0.19 ng/mL, with sensitivity 67.35% and specificity 75.53% for the diagnosis of BM [34]. Findings of clinical studies evaluating the role of PCT in patients with known or suspected meningitis are summarized in Table 1. Quantitative measures, including AUC, sensitivity, specificity, and cut-off points, reported in clinical studies included in this review are summarized in Table 2.

## 4. Discussion

Procalcitonin, a precursor of calcitonin, is a 116 amino acid peptide and a member of the calcitonin superfamily of peptides, with a molecular weight of 14.5 kDa. PCT is synthesized by the parafollicular C cells of the thyroid gland and is involved in calcium homeostasis. In addition, PCT is also produced by the neuroendocrine cells of the lung and the intestine. In the CNS, cells likely to be sources of CSF PCT include the neurons, astrocytes, and microglia in the parenchyma and meningeal cells. Normal S–PCT concentration is <0.05 ng/mL, with a reported half-life of 25–30 h [35]. Procalcitonin is considered a sensitive and specific marker of certain bacterial infections, such as pneumonia, meningitis, and pyelonephritis, and has been used as tool for the assessment of disease severity. In addition to bacterial infections, increased PCT levels have been identified in other clinical conditions, including severe fungal infections, trauma, burns, major surgery, and medical therapy that stimulates cytokine production. In these cases, S–PCT levels are less elevated and rarely exceed 0.5 ng/mL [36]. There are several hypotheses regarding the pathophysiology of PCT; most suggesting that procalcitonin may be involved in calcium metabolism, cytokine network, and the modulation of nitric oxide (NO) synthesis [37]. In sepsis, PCT hypersecretion emanates from multiple tissues throughout the body, which are not traditionally viewed as endocrine tissues. It is likely that PCT in sepsis potentiates the inflammation cascade by increasing leukocyte-derived cytokines and augmenting reactive oxygen species [38]. PCT secretion is stimulated in bacterial infections by various cytokines, such as IL–1, IL–6, and tumor necrosis factor-alpha. In contrast, PCT production is down-regulated in viral infections, probably due to increased interferon gamma production. Consequently, PCT is considered a useful tool for diagnosing sepsis and repeat S–PCT measurements over time can be used to monitor response to therapy. Most currently used inflammatory markers do not reliably differentiate between a systemic inflammatory response and sepsis. However, because PCT is, generally, not induced by severe viral infections or non-infectious inflammatory reactions, PCT can help distinguish bacterial from viral infections and differentiate between infectious and non-infectious origins of systemic inflammatory response syndrome (SIRS), acute respiratory distress syndrome (ARDS), pancreatitis, cardiogenic shock, and acute rejection of transplanted organs [39].

In CNS infections, disruption of the blood brain barrier (BBB) has been documented in patients with bacterial infections and in experimental models [40,41]. Elevated CSF PCT levels in bacterial meningitis patients seem to be the result of this mechanism and some studies have shown higher CSF PCT levels in patients with Gram-negative bacteria compared to patients with Gram-positive bacteria [42]. In CNS infection cases, microglia and meningeal cells express the responding receptors (Toll-like receptors [TLRs]) to the invading bacteria [43]. Several questions regarding PCT synthesis and secretion by brain cells during bacterial meningitis have not been resolved and need further investigation.

Prompt diagnosis and appropriate antimicrobial treatment are of paramount importance and can contribute to a reduced morbidity and mortality in sepsis. Procalcitonin is an acute-phase protein with faster kinetics than C-reactive protein (CRP) and erythrocyte sedimentation rate (ESR), and is detectable in serum within 4–6 h after the onset of a bacterial infection. PCT serum levels peak within 24 h and start to decline by approximately 50% daily with effective treatment [44,45,46]. Although there is no “gold standard” for the diagnosis of most infections, several biomarkers have been used as tools to monitor disease progression. An ideal marker should help with early diagnosis and therapeutic decision-making in bacterial infections and should also help clinicians assess the course and prognosis and, in that regard, PCT seems to be superior compared to other commonly used biomarkers.

Despite advances in diagnosis and treatment, bacterial meningitis is a neurological emergency, requires treatment in a high acuity care unit, and remains an important cause of mortality. The diagnosis and management of bacterial meningitis requires various biological tests and a multidisciplinary approach. Empiric antimicrobial and adjunctive therapy should start as soon as there is clinical suspicion of meningitis. Regarding laboratory findings, a left shift in peripheral white blood cell count, elevated serum PCT and C-reactive protein, CSF pleocytosis with predominance of polymorphonuclear leukocytes, and decreased glucose concentration are predictive of bacterial meningitis. CSF analysis is a gold standard for the diagnosis of meningitis: CSF gram staining reveals bacteria in 50% to 80% of cases and cultures are positive in 80% of cases, at best. However, the sensitivity of both tests is <50% in patients already receiving antibiotics. CSF leukocyte count, protein, glucose and lactate concentration, and a latex agglutination test adapted for the rapid direct detection of soluble bacterial antigens in CSF lack the specificity and sensitivity for the diagnosis of meningitis and can only define a clinical probability. In fact, the relatively imprecise nature of the cutoff values for these markers can make their interpretation difficult. Furthermore, in the early phases of acute bacterial and viral meningitis, differential diagnosis is difficult because signs and symptoms are often non-specific and, therefore, differentiation between bacterial and viral meningitis remains a difficult problem for clinicians. Biomarkers, like CRP, procalcitonin, or sTREM–1, may be useful for diagnosis and can help differentiate between viral and bacterial meningitis [47,48]. Serum and CSF PCT levels can be more useful in the diagnosis of bacterial meningitis and in distinguishing bacterial from viral meningitis. A systematic review published by Markanday in 2015 compared serum PCT versus CRP as markers for bacterial infection and showed that, compared to CRP, S–PCT had a higher sensitivity and specificity for differentiating bacterial from noninfectious causes of inflammation [49].

Diagnosis of meningitis requires a detailed history and physical examination combined with high clinical suspicion and appropriate cultures. Prognosis of meningitis depends on rapid diagnosis, identification of the cause, and prompt implementation of appropriate antibiotic treatment. Because clinical and laboratory data available within a few hours after hospital admission are not reliable (except for when bacteria are found in CSF under the microscope), use of biological markers has been proposed as a tool to improve the accuracy of initial diagnosis and, in this setting, serum and CSF procalcitonin measurements seem to be of great value. Because of its high specificity and positive predictive value, elevated S–PCT concentrations (>0.5 ng/mL) indicates ongoing and, potentially, severe systemic infection. Because C-reactive protein is the inflammatory marker most widely used in emergency departments to discriminate bacterial from viral infections, Gerdes et al. published a meta-analysis in 1998 from 35 studies aiming to assess the usefulness of CRP in discriminating bacterial from viral meningitis. The meta-analysis showed that, although the majority of authors propose using CRP as an additional tool for discriminating bacterial from viral meningitis, only negative CRP tests are highly informative in most clinical settings [50]. However, procalcitonin seems to be a valuable tool for discriminating the causative factor of meningitis as, since 1997 and 1998, two French studies showed that, using a cut-off range of 0.5–2 ng/mL, S–PCT had 100% sensitivity and specificity in discriminating bacterial from viral meningitis [51]. Similarly, a more recent meta-analysis published by Vikse et al. in 2015 showed that procalcitonin has a 90% sensitivity and a 98% specificity in the discrimination between bacterial and viral meningitis [26].

This review aims to provide clinicians with an overview of the role of S–PCT and CSF-PCT as diagnostic markers in CNS infections. Several publications assessing the role of PCT as a guide for antibiotic therapy in adult meningitis patients suggest that S–PCT is a sensitive, specific, and prognostic marker of bacterial infections, therefore, S–PCT and CSF PCT measurement can help differentiate bacterial from viral meningitis. Interpretation of PCT levels must take into consideration the clinical presentation of the CNS infection. In addition, knowledge of assay characteristics is important for setting specific cut-off values and functional assay sensitivities. Most clinical studies presented in this review have limitations, including small sample sizes and inconsistent reporting of laboratory findings: In some, AUC values and cut-off values were not reported, while a few were published in languages other than English and, therefore, we only included in the review data reported in the Abstract. Therefore, even though S–PCT seems to be a useful biomarker for the diagnosis and possible prognosis in patients with BM, additional data from larger, well-designed studies are needed to better evaluate the role of procalcitonin in the differentiation between viral and bacterial meningitis and as tool to improve the overall management of patients with meningitis.

## 5. Conclusions

Serum PCT is a biomarker with high sensitivity and specificity in identifying patients with sepsis and can be useful for the diagnosis of bacterial infections. This literature review identified several studies evaluating the role of S–PCT in the assessment of patients with central nervous system infections. Published data suggests that, compared to other acute phase biomarkers, S–PCT is superior as a sepsis biomarker in acute meningitis and can help differentiate bacterial from viral meningitis. Combined with good clinical judgment and appropriate use of antimicrobial agents, S–PCT could be a valuable adjunct in the timely diagnosis and management of sepsis in patients with CNS infection.

## Figures and Tables

**Table 1:** Clinical studies evaluating the role of procalcitonin (PCT) in patients with known or suspected meningitis.

Reference	Origin	Study Design	Patient Population	Findings
[3]	St. Etienne, France	Prospective study	105 pts (23 with BM, 57 with VM, 25 controls), 54 women, 51 men, mean age 42 years (range 16–82 years)	S–PCT was the best marker for differentiating BM vs. VM. with S–PCT > 0.2 ng/mL as the cutoff, S–PCT sensitivity and specificity approaches 100% for diagnosing acute BM
[4]	Heidelberg, Germany	Prospective case series	30 pts (13 men, 17 women), mean age 52 (range, 16–87 years)	S–PCT is useful for distinguishing BM from VM. Increased S–PCT levels have a high specificity for bacterial infection
[5]	St. Etienne, France	Prospective study	179 patients with suspected meningitis: 32 patients with BM, 90 patients with VM, 57 patients did not have meningitis	S–PCT is the most discriminant variable for differentiating BM vs. VM, with a threshold value 0.93 ng/mL
[6]	Saitama, Japan	Prospective study	42 patients requiring CSF examination, 12 patients with non-inflammatory CNS disease as controls, 22 men, mean age 37.8 years; 20 women, mean age 38.1 years	CSF PCT levels not significantly different in BM, VM, or mycotic meningitis. S–PCT > 0.1 mcg/L in all BM patients. AUC and cut-off values were not reported
[7]	Berlin, Germany	Case series, 12 adults with meningitis	12 pts: 7 men, 5 women, mean age 48.6 years	S–PCT has a limited diagnostic value for BM in adults
[9]	Ljubljana, Slovenia	Prospective study, 45 adults with CNS infection	20 BM patients: 11 men, 9 women, mean age 55 years; 25 TBE patients: 13 men, 12 women, mean age 49 years	Median S–PCT 6.45 ng/mL (0.25–43.76) in patients with BM vs. 0.27 ng/mL (0.05–0.44) in patients with TBE. S–PCT and CSF PCT > 0.5 ng/mL is a reliable indicator of BM
[10]	Dresden, Germany	Prospective ICU study	11 ventriculitis patients with negative CSF (6 men, 5 women, mean age 44.3 years), 4 ventriculitis patients with positive CSF (2 men, 2 women, mean age 56.2 years), 10 community BM (7 men, 3 women, mean age 49.4 years)	S–PCT alone is not helpful for diagnosis of ventriculitis
[11]	Munich, Germany	Case report: 73 year old woman, post-myelogram chemical meningitis	7 ICU BM patients (4 men, 3 women, mean age 55 years), and one woman with aseptic, chemical meningitis	S–PCT is useful in differentiating bacterial vs. chemical CNS inflammation
[12]	Poland	Observational study, 33 adult patients	17 bacterial meningoencephalitis patients vs. 16 lymphocytic meningitis patients	CSF PCT and S–PCT significantly higher in BM vs. lymphocytic meningitis (CSF PCT 0.63 ng/mL vs. 0.23 ng/mL, *p* < 0.05, S–PCT 9.97 ng/mL vs. 0.27 ng/mL, *p* < 0.01
[13]	St. Etienne, France	Prospective study, 48 BM patients with S–PCT > 0.5 ng/mL on admission	48 BM patients: 21 men, 27 women, mean age 55 years	S–PCT levels declined rapidly with antibiotic therapy
[14]	Borgsdorf, Germany	Prospective Study	40 patients with AD, 12 with FTD, 8 with DLB, 12 with VD, 16 with acute neuroinflammation, 50 controls	Measured S–PCT, CSF PCT. S–PCT elevated in meningitis. CSF PCT helpful in diagnosing dementia
[15]	Hvidovre, Denmark	Observational cohort study	52 patients (25 men, 27 women) with suspected meningitis, median age 36 (range, 13–92 years)	S–PCT has a moderate overall accuracy for differentiating BM vs. non-bacterial disease
[16]	Paris, France	Prospective multicenter study, 151 meningitis patients	133 NBM patients: 66 men, 67 women, mean age 33 years vs. 18 BM patients: 9 men, 9 women, mean age 52 years	Serum CRP and S–PCT are excellent predictors of BM
[17]	St. Etienne, France	Prospective study, patients with negative CSF exam	35 patients (17 men, 18 women, mean age 55 years) with BM, 218 patients (116 men, 102 women, mean age 35 years) with VM	S–PCT had very high diagnostic value for distinguishing BM vs. VM
[18]	Ahvaz, Iran	Prospective study, 36 patients with acute meningitis	36 acute meningitis patients: 26 men, 10 women, mean age 38.4 years	S–PCT levels were reduced after 72 h in patients who improved, but remained higher in patients who did not improve
[19]	Seoul, Korea	Prospective study, 78 postoperative meningitis patients	14 patients with BM: 4 men, 10 women, median age 52 (range 44–63) vs. 64 aseptic meningitis patients: 35 men, 29 women, median age 47.5 (range 35–61 years)	S–PCT has limited value for diagnosing BM (50% sensitivity, 80% specificity for S–PCT ≥ 0.15 ng/mL).
[20]	Guangzhou, China	Retrospective study, NICU pts	22 sepsis patients (16 men, 6 women, mean age 58 years), 22 severe sepsis patients (17 men, 5 women, mean age 55.4 years), 12 septic shock patients (5 men, 6 women, mean age 51.9 years), and 48 SIRS patients (28 men, 20 women, mean age 51.8 years)	Assessed S–PCT for discrimination between sepsis and SIRS. S–PCT levels were significantly different between groups at all stages of sepsis. S–PCT has value for discriminating sepsis from SIRS and for determining sepsis severity
[21]	Cairo, Egypt	Prospective study, 40 patients with suspected acute meningitis	16 ABM patients (9 men, 7 women, mean age 39 years) and 24 patients with acute ASM (21 men, 3 women, mean age 29 years), 10 controls (7 men, 3 women, mean age 40 years)	S–PCT significantly higher in BM compared to ASM patients (2.49 ± 2.54 vs. 0.89 ± 0.69, *p* < 0.001). Difference in BM vs. ASM persisted after 3 days of therapy
[22]	Nanjing, China	Prospective observational study, 120 patients	45 BM patients (30 men, 15 women, mean age 50) vs. 75 non-BM patients (55 men, 20 women, mean age 47 years)	S–PCT and CSF PCT levels increase in patients with BM
[23]	Abu Dhabi, UAE	Prospective, observational study	36 head trauma patients with EVD (30 men, 6 women, mean age 32.8 years)	High S–PCT in patients with ventriculostomy-related infections. Early S–PCT increase is a valid indicator of bacterial CNS infection
[24]	Beijing, China	Retrospective study, 178 post-neurosurgical patients	50 with PNBM (23 men, 27 women, median age 42 years) vs. 128 without PNBM 49 men, 79 women, median age 42 years)	CSF lactate and PCT have significant diagnostic value for PNBM and could be useful in differentiating PNBM from non-PNBM
[25]	Alexandroupolis, Greece	Prospective, observational study, 58 patients	19 BM patients (12 men, 7 women, mean age 41 years) vs. 11 VM patients (8 men, 3 women, mean age 24 years) vs. 28 controls (20 men, 8 women, mean age 30 years)	CSF–PCT is helpful in distinguishing BM from VM and other noninfectious CNS diseases
[27]	Busan, Korea	Retrospective study of patients with TBM who had S–PCT measured	26 TBM patients (13 men, 13 women, mean age 57 years) vs. 70 BM patients (42 men, 28 women, mean age 64 years) vs. 49 VM patients (24 men, 25 women, mean age 40 years)	S–PCT levels not significantly different in TBM vs. VM, but S–PCT > 0.4 ng/mL was a predictor of poor prognosis in TBM
[29]	Toledo, Spain	Prospective, observational study	220 meningitis patients (136 men, 84 women, mean age 30 years)	PCT has high diagnostic powers and outperforms CRP and leukocytes for the detection of bacterial meningitis
[28]	Toledo, Spain	Prospective observational study	154 ED patients over age 15	Logistic regression shows that S–PCT ≥ 0.8 ng/mL + CSF lactate ≥ 33 mg/dL are strong predictors of BM (*p* < 0.001)
[31]	Toledo, Spain	Prospective, observational study	98 ED patients (66 men, 32 women, mean age 44 years), 38 BM patients (mean age 61.5 years), 33 VM patients (mean age 36 years) 15 with probable VM, but negative cultures, 12 with antibiotic treatment and negative cultures	S–PCT higher in BM vs. VM (11.47 ± 7.76 vs. 0.10 ± 0.15 ng/mL, *p* < 0.001). S–PCT is superior to S-CRP for BM detection
[32]	Jilin, China	Prospective observational study	66 meningitis patients: 37 men, 29 women, mean age 40.21 years (24 suppurative meningitis, 20 VM, 22 TBM), 20 controls: 11 men, 9 women, mean age 43.05 years	S–PCT is significantly higher in suppurative meningitis, declined significantly after 72 h and 7 days of treatment. CSF PCT is significantly lower in VM compared to TBM and suppurative meningitis.
[33]	Busan, Korea	Retrospective study, suspected meningitis patients	80 patients with BM (49 patients, 31 women, median age 66 years) vs. 58 VM patients (30 men, 28 women, median age 37 years)	S–PCT levels >0.12 ng/mL are significant marker for differentiating BM vs. VM
[34]	Xi’an, China	Prospective observational study, 143 ICU patients with CNS disease	49 BM patients (36 men, 13 women, median age 43 years) vs. 25 TBM patients (15 men, 10 women, median age 42 years) vs. 34 VM patients (25 men, 9 women, median age 39.5 years) vs. 15 AIE patients (6 men, 9 women, median age 27 years) vs. 20 NINSD (11 men, 9 women, median age 43 years)	CSF PCT levels were significantly higher in BM compared to TBM, VM, AIE, or NINSD

ABM = Acute Bacterial Meningitis, AD = Alzheimer’s disease, AIE = Autoimmune Encephalitis, ASM = Aseptic Meningitis, AUC = Area Under the Curve, AUROC = Area Under The Receiver Operating Characteristic Curve, BM = Bacterial Meningitis, CI = Confidence Interval, CRP = C-reactive protein, CSF = cerebrospinal fluid, DLB = Dementia with Lewy bodies, ED = Emergency Department, EVD = External Ventricular Drainage, FTD = Frontotemporal dementia, LP = Lumbar Puncture, NBM = Nonbacterial Meningitis, NICU = Neurological Intensive Care Unit, NINSD = Non-Inflammatory Nervous System Disease, NPV = Negative Predictive Value, PMN = Polymorphonuclear, PNBM = Post-Neurosurgical Bacterial Meningitis, PPV = Positive Predictive Value, Pts = patients, S–PCT = Serum Procalcitonin, SIRS = Systemic Inflammatory Response Syndrome, Tb = Tuberculosis, TBE = Tick-Borne Encephalitis, TBM = Tuberculous Meningitis, VD = Vascular Dementia, VM/E = Viral Meningitis/Encephalitis, VM = Viral Meningitis.

**Table 2:** Area under the Curve (AUC), sensitivity, specificity, and cut-off points reported in clinical studies evaluating Serum Procalcitonin (S–PCT) or Cerebrospinal Fluid Procalcitonin (CSF PCT) in patients with known or suspected meningitis.

Reference	Biomarker	AUC (95% CI) *p* Value	Cut-off Point	Sensitivity	Specificity	Comments
[3]	S–PCT	Not reported	0.2 ng/mL	100%	100%	CSF PCT found only in two patients with hemorrhage. S–PCT sensitivity and specificity approach 100% for diagnosing acute BM
[4]	S–PCT	Not reported	0.5 ng/mL	69% (41–89%)	100% (79–100%)	S–PCT is a useful variable for distinguishing BM from VM. Increased S–PCT levels have a high specificity for bacterial infection
[5]	S–PCT	Not reported	0.93 ng/mL	Not reported	100%	S–PCT is the most discriminant variable for differentiating BM vs. VM
[7]	S–PCT	Not reported	1 ng/mL	58.3%	Not reported	S–PCT has limited diagnostic value in adults with BM
[9]	CSF PCT S–PCT	Not reported	0.5 ng/mL 0.5 ng/mL	55% 90%	100% 100%	Median S–PCT is significantly higher in BM (6.45 ng/mL range 0.25–43.76) vs. TBE (0.27 ng/mL, 0.05–0.44). S–PCT, CSF PCT > 0.5 ng/mL is a reliable indicator of BM
[10]	S–PCT	Not reported	1.0 ng/mL	100%	83%	S–PCT is not helpful for diagnosis of ventriculitis, but is highly diagnostic for BM
[11]	S–PCT	Not reported	1.0 ng/mL	Not reported	Not reported	S–PCT is useful in differentiating bacterial vs. chemical causes of CNS inflammation
[12]	CSF-PCT S–PCT	Not reported	Not reported	Not reported	Not reported	CSF PCT and S–PCT are significantly higher in bacterial meningoencephalitis vs. lymphocytic meningitis
[13]	S–PCT	Not reported	Not reported	Not reported	Not reported	Rapid S–PCT decline with antibiotic therapy reduces the value of repeating LP to assess antibiotic therapy effectiveness
[14]	CSF-PCT	0.83 (0.76–0.91), *p* < 0.0001	57 pg/mL 65 pg/mL	75% 63.9%	80% 90%	S–PCT levels are elevated in meningitis, but not in dementia
[15]	S–PCT	0.93 (0.64–0.99)	0.25 ng/mL	0.90 (0.55–1.0)	0.92 (0.62–1.0)	S–PCT is helpful for differentiating BM vs. VM, with an overall moderate accuracy (AUC 0.75, 0.50–0.89)
[16]	S-CRP S–PCT	0.81 (0.58–0.92) 0.98 (0.83–1.00)	22 mg/L 2.13 ng/mL	78% 87%	74% 100%	S-CRP and S–PCT are excellent predictors of BM
[17]	S–PCT	0.99 (0.99–1.00)	0.28 ng/mL	97%	100%	S–PCT: High diagnostic value for distinguishing BM vs. VM
[18]	S–PCT	Not reported	Not reported	Not reported	Not reported	S–PCT can be a useful marker for the follow up of BM patients
[19]	S–PCT	0.617	0.15 ng/mL	50%	80%	S–PCT is of limited value for diagnosing BM
[20]	S–PCT	0.799 (0.711–0.887)	2 ng/mL	75%	83.3%	S–PCT has value for discriminating sepsis vs. SIRS and determining sepsis severity
[21]	S–PCT	AUC shown, but value not reported	1.2 ng/mL	68.8%	83.3%	Admission S–PCT is higher in BM patients compared to ASM patients (2.49 ± 2.54 vs. 0.89 ± 0.69, *p* < 0. 001). The difference in BM vs. ASM persisted after 3 days of therapy (1.70 ± 1.58 vs. 0.64 ± 0.51, *p* < 0.001)
[22]	S–PCT	0.96 (0.93–1.00)	0.88 ng/mL	87%	100%	S–PCT and CSF PCT increased in BM patients.
[23]	S–PCT	Not reported	Not reported	Not reported	Not reported	Mean S–PCT < 2.0 ng/mL in patients with negative vs. 4.18 ng/mL in patients with positive CSF cultures
[24]	CSF PCT CSF lactate	0.746 *p* < 0.001 0.943 *p* < 0.001	0.075 ng/mL 3.45 mmoL/L	68.0% 90%	72.7% 84.4%	CSF lactate and CSF PCT have significant diagnostic value for PNBM
[25]	CSF PCT	Not reported	Not reported	100%	96.4%	CSF PCT 4.71 ± 1.59 ng/mL in BM, 0.13 ± 0.03 ng/mL in VM, <0.1 in patients with non-infectious disease
[27]	S–PCT	0.876 (0.688–0.972)	1.27 ng/mL	96.2% (80.4–99.9)	62.9% (50.5–74.1)	S–PCT useful for distinguishing TBM from BM. S–PCT > 0.4 ng/mL is predictor of poor outcome in TBM
[29]	S–PCT	0.972 (0.946–0.998)	0.52 ng/mL	93%	86%	Sensitivity 96%, Specificity 75% for ages >75 years
[28]	S–PCT + CSF lactate	0.992 (0.979–1.000, *p* < 0.001)	S–PCT ≥ 0.8 ng/mL + CSF lactate ≥33mg/dL	99%	98%	The combination of S–PCT ≥ 0.8 ng/mL + CSF lactate ≥ 33 mg/dL has excellent diagnostic value for predicting BM
[31]	S–PCT	0.965 (0.921–1), *p* < 0.001	1.1 ng/mL	94.6%	72.4%	S–PCT significantly higher in BM vs. VM (11.47 ± 7.76 vs. 0.10 ± 0.15 ng/mL, *p* < 0.001). Concluded that S–PCT is superior to serum CRP for detecting BM
[32]	S–PCT	Not reported	Not reported	Not reported	Not reported	S–PCT is significantly higher in suppurative meningitis and declined 72 h and 7 days after treatment
[33]	S–PCT	Not reported	0.12 ng/mL	88.75%	74.14%	S–PCT > 0.12 ng/mL is useful for differentiating BM vs. VM. S–PCT > 7.26 ng/mL associated with higher mortality (OR = 9.09, 95% CI: 1.74–47.12, *p* = 0.016)
[34]	CSF PCT	0.881 (0.810–0.932)	0.15 ng/mL	69.39	91.49%	CSF PCT levels are the most useful biomarker for the diagnosis of BM
[34]	S–PCT	0.759 (0.669–0.849)	0.19 ng/mL	67.35%	75.53%	S–PCT is less useful than CSF PCT for the diagnosis of BM
